# Expression of chemokine receptor CXCR4 in nasopharyngeal carcinoma: pattern of expression and correlation with clinical outcome

**DOI:** 10.1186/1479-5876-3-26

**Published:** 2005-06-26

**Authors:** Na Wang, Qiu-Liang Wu, Yan Fang, Hai-Qiang Mai, Mu-Sheng Zeng, Guo-Ping Shen, Jing-Hui Hou, Yi-Xin Zeng

**Affiliations:** 1 State Key Laboratory of Oncology in Southern China; 2 Department of Experimental Research, Cancer Center, Sun Yat-sen University, Guangzhou 510060, China; 3 Department of Pathology, Cancer Center, Sun Yat-sen University, Guangzhou 510060, China; 4 Department of Nasopharyngeal Carcinoma, Cancer Center, Sun Yat-sen University, Guangzhou 510060, China

**Keywords:** chemokine, CXCR4, immunohistochemistry, nasopharyngeal carcinoma

## Abstract

Nasopharyngeal carcinoma (NPC) is a tumor derived from epithelial cells and Epstein-Barr virus infection has been reported to be a cause of this disease. Chemokine receptor CXCR4 was found to be involved in HIV infection and was highly expressed in human malignant breast tumors and the ligand for CXCR4, CXCL12 (SDF-1), exhibited high expression in organs in which breast cancer metastases are often found. The metastatic pattern of NPC is quite similar to that of malignant breast tumors. In this study, we investigated the expression of CXCR4 in nasopharyngeal carcinoma (NPC) tissues by immunohistostaining. We found different staining patterns, which included localization in the nucleus, membrane, cytoplasm or a combination of them. The staining intensity was also variable among samples. The metastatic rates in patients with high compared to low or absent expression was 38.6% versus 19.8%, respectively (*P *= 0.004). High expression of CXCR4 was associated with poor overall survival (OS = 67.05% versus 82.08%, *P *= 0.0225). These results suggest that CXCR4 may be involved in the progression of NPC and that a high level of CXCR4 expression could be used as a prognostic factor.

## Introduction

Nasopharyngeal carcinoma (NPC) is a tumor derived from epithelial cells located in the posterior part of the nasopharynx. The nasopharynx has an abundant supply of regional lymphatic vessels, which drain along the internal jugular vein and the posterior cervical and retropharyngeal chains. As a result, NPC frequently spreads regionally leading to early lymph-node involvement in the neck. Systemic dissemination also occurs more readily than in other head-and-neck cancers, frequently involving bones, lung, and liver [1]. Although the primary tumor is sensitive to radiotherapy, NPC-related deaths occur because of secondary spread of tumor cells. It has been observed that at the time of diagnosis, 60–85% of NPC patients already have clinically detectable metastases in the regional lymph nodes or distant organs such as the lungs and bone [2]. Since there is no effective treatment for NPC at the stage of metastasis, the prognosis remains poor with a 5-year survival around 50%[3].

The metastatic process results from several sequential steps and represents a highly organized, non-random, organ-selective process [4]. Muller et al [5] found that the chemokine receptor CXCR4 and CCR7 were highly expressed in human malignant breast tumors compared to normal breast tissue. The ligands for these receptors – CXCL12 (SDF-1) for CXCR4 and CCL21 for CCR7 – exhibit high expression in organs in which breast cancer metastases are often found. The organ tropism of metastatic NPC often recapitulates that observed in malignant breast tumors. In addition, Staller et al [6] observed an inverse correlation between CXCR4 expression and survival in patients with renal cell carcinoma (RCC). They also noted that the von Hippel-Lindau tumor suppressor protein pVHL negatively regulates CXCR4 expression owing to its capacity to target hypoxia-inducible factor (HIF) for degradation under normoxic conditions. The process is suppressed under hypoxic conditions, resulting in HIF-dependent CXCR4 activation. Interestingly, the vHL gene is located at 3p26-3p25. This region displays loss of heterozygosity in NPC and the loss of 3p26-13 has been associated to early events in the carcinogenesis of NPC [7,8].

The chemokine receptor CXCR4 is the only physiological receptor for SDF-1 (a member of the CXC subfamily of chemokines) [9-11]. Chemokines represent a large family of about 50 proteins that modulate cell trafficking and angiogenesis, during infection and inflammation and play a significant role in the tumor microenvironment. [12].

CXCR4 plays a major role in embryogenesis, homeostasis and inflammation and can function as a coreceptor for T lymphocytotrophic HIV-1 isolates [13,14]. CXCR4 is also the only chemokine receptor which mRNA expression is regulated during trophoblast differentiation in vitro [15]. Kobayashi et al reported that CXCR4 was down-regulated during differentiation of viral antigen-specific CD8 (+) T cells and could be used to distinguish subsets of CD8 (+) T [16].

CXCR4 expression has been reported in several epithelial, mesenchymal and haematopoietic cancers [17]. Recently CXCR4 has been shown to be expressed by tumor cells in breast cancer, non-small cell lung cancer, pancreatic cancer, prostate cancer, thyroid cancer and to play an important role in their metastatic process [5,18-22]. Finally, Xu Y et al found that CXCR4 was highly expressed in NPC cell lines, and its expression was associated with differentiation grade and proliferation ability of NPC cells [23].

Therefore, we asked whether CXCR4 expression is associated with the prognosis and differentiation of NPC. Considering its potential effect on the development of metastases, we investigated the expression of CXCR4 in NPC tissue by immunohistochemistry and observed that high CXCR4 expression is associated with poor survival independently of the differentiation status in NPC.

## Materials and methods

### Reagents and antibodies

The anti-CXCR4 mouse monoclonal antibody (MAB 172; R&D Systems; dilution 1:600) was used for immunohistochemical analysis. The PV-9000 Polymer Detection System was used for immunohistochemical staining according to the manufacturer's recommendations (Golden Bridge International, USA).

### Nasopharyngeal carcinoma tissues

Immunohistochemistry was performed on undifferentiated NPC carcinomas tissues collected from 194 patients who were admitted to the Sun Yat-sen University Cancer Center in the year 2000 and could be followed for three years until 2004. We also analyzed 26 NPC tissue samples of different histological subtype containing 10 undifferentiated carcinomas, 10 differentiated carcinomas and 6 keratinising squamous cell carcinomas from these patients admitted to the center from 2000 to 2004. All samples were obtained with full patient consent.

### Immunohistochemical analysis

Samples were fixed in 4% paraformaldehyde or 10% formalin and embedded in paraffin. Four mm sections were cut and placed on silane-coated slides for immunohistochemical studies. Part of the specimens was stained with H&E and microscopically examined to confirm the diagnosis. The paraffin sections were dewaxed and pretreated in 0.01 M sodium citrate buffer (pH 6.0) for 15 min at 95°C to unmask tissue antigen. These sections were then incubated with 3% hydrogen peroxide for 30 minutes at room temperature to block endogenous peroxidase. Immunostaining was performed with anti-CXCR4 antibody (dilution 1:600) at 4°C overnight. The sections were developed according to manufacturer's recommendations (PV-9000 Polymer Detection System, Golden Bridge International, USA) and counterstained with hematoxylin. CXCR4 positivity was graded semiquantitatively according to Carcangiu's method[24] as negative or weak (total score ≤ 3) and positive (total score ≥ 4) by two independent investigators without knowledge of the patients' clinicopathological features and the clinical follow-up data.

### Statistical analysis

Survival was calculated by the Kaplan-Meier method, and the resulting curves were compared using the log-rank test. Fisher's exact test and the χ^2 ^test were used to analyze the association between two categorical variables. *P *<0.05 was considered to be statistically significant.

## Results

### Expression pattern of CXCR4 in nasopharyngeal carcinoma

**Figure 1:**
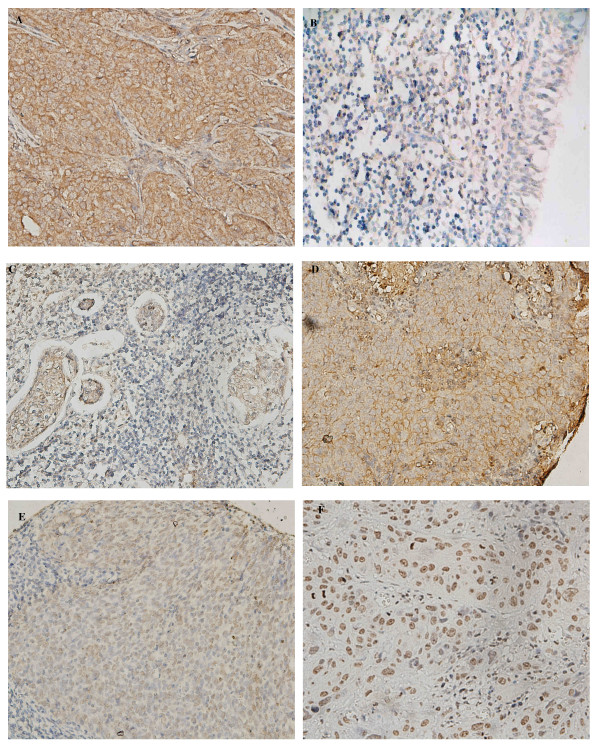
Immunohistochemical evaluation of CXCR4 expression. (A) Cytoplasm and membrane staining in a breast cancer tissue as a positive control. (B) Weak or absent staining in a normal nasopharyngeal epidermis. (C) An undifferentiated nasopharyngeal carcinoma of cytoplasm staining with adjacent lymphocytes displaying predominant cytoplasmic staining. (D) An undifferentiated nasopharyngeal carcinoma of membrane staining. (E) An undifferentiated nasopharyngeal carcinoma of nucleus staining. (F) Representative nucleus staining in a keratinising squamous cell carcinoma of nasopharynx. (All of the photomicrographs are high-powered magnified, ×400).

We first examined 194 NPC cases for which three-year follow up information was available. All the tumors were pathologically classified as WHO type III (undifferentiated carcinoma). We observed different staining patterns of CXCR4 in NPC tumor cells: 135 showed nucleus staining, 18 nucleus and cytoplasm staining, 2 nucleus and cytoplasm and membrane staining, 14 cytoplasm staining, 22 cytoplasm and membrane staining, 2 membrane staining and one negative staining (Fig. 1, C, D and 1E). Since positive nucleus staining was the most frequently observed pattern, we examined whether there was a correlation between positive nucleus staining and survival by dividing samples into two categories according to positive or negative nuclear staining. No statistically significant correlation was noted with survival. In addition, nuclear staining was not related to intensity of staining.

Intensity of staining was also observed to be variable among samples ranging from absent or weak to strong (Fig. 1, C, D, E). The non-cancerous nasopharyngeal epidermis stained either weakly or not at all (Fig. 1, B). Strong CXCR4 expression was detected in 45.4% (88/194) of the cancerous samples while the remaining displayed weak (105 samples) or negative (1 sample) staining. Among the samples with strong CXCR4 expression 67 showed nuclear staining, 2 nuclear and cytoplasm staining, 6 cytoplasm staining, 11 cytoplasm and membrane staining, and 2 membrane staining. The distribution of the CXCR4 staining is summarized in Table 1.

**Table 1:** Staining location of CXCR4 in the 194 undifferentiated nasopharyngeal carcinomas

Expression Pattern Total (n = 194)	Nucleus staining	Nucleus and cytoplasm staining	Nucleus and cytoplasm and membrane staining	Cytoplasm staining	Cytoplasm and membrane staining	Membrane staining	Absent staining
Strong staining (n = 88)	67	2	0	6	11	2	0
Weak or absent staining (n = 106)	68	16	2	8	11	0	1

### CXCR4 expression is correlated with metastatization and tumor-specific survival

Next, we analyzed the relationship between distant metastases and CXCR4 staining. Metastatic rate was significantly higher in patients with strong CXCR4 (38.6%) compared to weak or absent (19.8%) staining (*P *= 0.004; Table 2). Distant metastatic sites included lungs, brain, liver and bone.

**Table 2:** Staining intensity of CXCR4 and clinical characteristics of NPC patients

Characteristics	Strong staining (n = 88)	Weak or absent staining (n = 106)	Total (n = 194)	***P***
Age				
Median (range)	45(25–70)	46(14–72)	45 (14–72)	
<50	58	65	123	0.408
≥50	30	41	71	
Gender				
Female	17	27	44	0.308
Male	71	79	150	
Stage				
I/II	21	39	60	0.052
III/IV	67	67	134	
N stage				
N0	18	25	43	0.601
non-N0	70	81	151	
Metastasis (follow-up)				
Yes	34	21	55	***0.004***
No	54	85	139	

**Figure 2:**
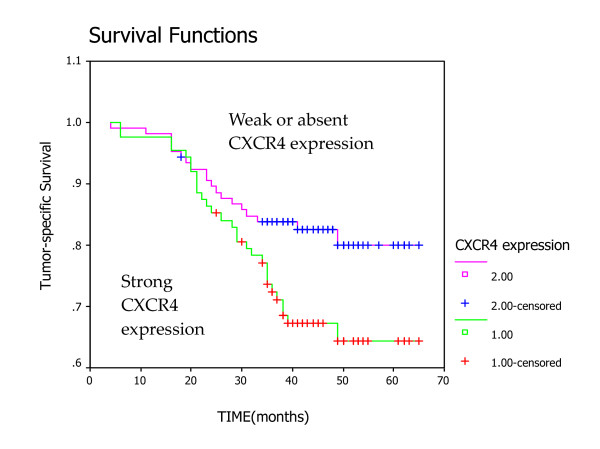
Kaplan-Meier analysis of tumor-specific survival in patients with NPC according to CXCR4 expression. Strong staining was associated with poor survival compared with weak or absent expression (OS = 67.05% versus 82.08%, *P *= 0.0225).

We further analyzed the intensity of CXCR4 staining in relation with patient survival at 3 years follow up. During this period, 28.4% (55/194) patients experience metastatic recurrence and the overall survival (OS) was 75.3% (146/194), expectedly higher than the reported 5-year survival rate for NPC [3]. Strong CXCR4 staining was associated with poor survival (OS = 67.05% versus 82.08%, *P *= 0.03) (Fig. 2) while no correlation was observed between level of CXCR4 expression and age, gender, or tumor stages (Table 2).

### Expression of CXCR4 is not related to the status of differentiation of NPC

CXCR4 has been reported to be involved in NPC cells differentiation [23]. Most NPC in high risk areas belong to WHO type III (undifferentiated carcinoma) while the more differentiated type, keratinising squamous cell carcinoma, and the differentiated carcinoma are rarely observed. To examine if CXCR4 expression is influenced by the differentiation status in NPC, we collected 26 NPC samples consisting of 6 keratinising squamous cell carcinomas, 10 differentiated carcinomas and 10 undifferentiated carcinomas. Different staining patterns consisted of 3 nuclear, 2 nuclear and cytoplasm, 13 nuclear, cytoplasm and membrane, 6 cytoplasm and 2 cytoplasm and membrane staining (Figure 1, F). The distributing of the CXCR4 location is summarized in Table 3. There was no correlation between staining location and differentiation status of the NPC samples. In addition, 34.6% (9/26) samples stained strongly while the other 17 samples stained weakly with no correlation with differentiation status.

**Table 3:** Staining location and intensity of CXCR4 in 26 NPC samples with different differentiation status

Expression Pattern Total (n = 26)		Nucleus staining	Nucleus and cytoplasm staining	Nucleus and cytoplasm and membrane staining	Cytoplasm staining	Cytoplasm and membrane staining
	Keratinising squamous cell Carcinoma (n = 3)	3	0	0	0	0
Strong staining (n = 9)	Differentiated carcinomas (n = 4)	0	0	1	2	1
	Undifferentiated carcinomas (n = 2)	0	0	2	0	0
	Keratinising squamous cell Carcinoma (n = 3)	0	1	2	0	0
Weak staining (n = 17)	Differentiated carcinomas (n = 6)	0	0	2	3	1
	Undifferentiated carcinomas (n = 8)	0	1	6	1	0

## Discussion

The chemokine receptor CXCR4 is the only physiological receptor for SDF-1. SDF-1/CXCR4 interactions have been intensively investigated in immunology, especially with regard to mechanism of HIV-1 infection to T lymphocytes [13,14]. Recently attention was turned to its involvement in cancer development and progression. Balkwill [17] reviewed that malignant cells from different types of cancer expressed CXCR4 and interact with its ligand SDF-1. Several other studies on breast, lung, pancreatic, prostate and thyroid cancer, and glioma suggested a role of CXCR4 in the metastasatic process [5,18-22].

In the present study, we evaluated CXCR4 expression in nasopharyngeal carcinoma by immunohistochemistry. The observed association between strong CXCR4 expression and poor tumor-specific survival suggests that CXCR4 expression levels influence the metastatic behavior of NPC. Among the known factors associated with NPC induction, Epstein-Barr virus (EBV) infection plays an important role. Latent membrane protein (LMP)-1 is the EBV-encoded protein with the most significant oncogenic properties. In addition, LMP-1 induces NF-κB activation which has important effects on EBV-infected cell survival [25]. NF-κB, in turn, regulates the motility of breast cancer cells by directly up-regulating the expression of CXCR4. Over expression of the inhibitor of NF-κB (IκB) in breast cancer cells constitutively expressing NF-κB results in reduced expression of CXCR4 and a corresponding loss of SDF-1α-mediated migration in vitro [26]. This observation may explain that the correlation between high expression of CXCR4 in NPC cells and metastatic rate in NPC patients which in turn affects their survival.

Our results are consistent with others' recent studies. Murakami et al [27] suggested that a limited number of chemokine receptors might play a critical role in determining organ-tropism in metastatic melanoma by regulating chemoattraction, adhesion, and survival. In particular, they advocated a role for chemokine receptor 7 (CCR7) in lymph node metastasis, CXCR4 in pulmonary metastasis, and CCR10 in skin metastasis. Moreover, CXCR4 expression in neuroblastoma primary tumors is significantly correlated with the pattern of metastatic spread. Similar findings were also reported from investigations on prostate cancer, head and neck squamous cell carcinoma and neuroblastoma primary tumors [28-30]. Furthermore, Lapteva et al found that small interfering RNA (siRNA) against CXCR4 effectively abrogated breast tumor growth in vivo implying CXCR4 as potential target to control breast cancer growth and metastasis [31].

We noted distinct patterns of CXCR4 staining in NPC tissues including nuclear, membrane and cytoplasm (Figure 1). CXCR4 is a serpentine transmembrane protein that mediates signal transduction according to its location on the cell membrane or in the cytoplasm. In this study, we observed a high percentage of nuclear staining of CXCR4. Three previous studies also identified nuclear localization of CXCR4 in hepatocellular carcinoma [32], invasive ductal mammary carcinoma [33] and non-small-cell lung cancer (NSCLC) [19]. Kato et al [33] reported nuclear staining of CXCR4 and defined the expression pattern of CXCR4 as diffuse or focal observing a significant correlation with the rate of lymph node metastases in breast cancers. Spano et al [19] found that strong CXCR4-nuclear staining was associated with significantly better outcome in early-stage NSCLC. However, we could not correlate nuclear staining with clinical outcome. This inconsistency of our findings with others' reports may be the result of the different tissue types studied and larger studies will be required to draw definitive conclusions.

To test whether a relationship exists between CXCR4 expression and differentiation of NPC, as reported by Xu et al [23], we collected another 26 NPC samples with different differentiation status. As shown in Table 3, we did not observe a correlation. Such correlation was also not fund in other solid tumor cells.

In summary, our results identified an aberrant expression of CXCR4 in NPC cells and the high level of CXCR4 expression correlated with distant metastasis and poor tumor-specific survival. These results further imply that CXCR4 could be involved in NPC progression and strong staining of CXCR4 could be used as a predictor for NPC prognosis.

## Authors' contributions

Yi-Xin Zeng is the general supervisor of the research group. He made substantial contributions to conception and designed the experiments, gave final approval of the version to be submitted.

Na Wang made fundamental contributions to this article. She completed most of the experiments, analysised the results and drafted this article.

Qiu-Liang Wu performed the evaluation of CXCR4 positivity.

Yan Fang made the tissue microarray and provided part of the samples.

Hai-Qiang Mai collected the other part of the samples.

Mu-Sheng Zeng helped to design the experiments.

Guo-Ping Shen performed the statistical analysis.

Jing-Hui Hou helped to do the experiments.
